# Assessment of effects of methylene blue on intestinal ischemia and reperfusion in a rabbit model: hemodynamic, histological and immunohistochemical study

**DOI:** 10.1186/s12917-020-02279-6

**Published:** 2020-02-12

**Authors:** Juan Morgaz, Sergio Ventura, Pilar Muñoz-Rascón, Rocio Navarrete, José Pérez, María del Mar Granados, José Andrés Fernández-Sarmiento, Juan Manuel Domínguez, Verónica Molina, Rafael J. Gómez-Villamandos, Rafael Zafra

**Affiliations:** 1 Department of Animal Medicine and Surgery, Faculty of Veterinary Sciences, University of Cordoba, Córdoba, Spain; 2 Faculty of Veterinary Sciences, University of Cordoba, Francisco Santisteban Hospital, Campus de Rabanales, 14014 Córdoba, Spain; 3 Department of Comparative Anatomy and Pathological Anatomy, Faculty of Veterinary Sciences, University of Cordoba, Córdoba, Spain; 4 Department of Animal Health, Faculty of Veterinary Sciences, University of Cordoba, Córdoba, Spain

**Keywords:** Intestinal ischemia reperfusion, Methylene blue, Cardiac output, Shock, Immunohistochemical damage, Rabbit

## Abstract

**Background:**

Intestinal ischemia-reperfusion (IR) is an important clinical occurrence seen in common diseases, such as gastric dilatation-volvulus in dogs or colic in horses. Limited data is available on the use of methylene blue in veterinary medicine for intestinal ischemia-reperfusion. The present study aimed to compare the hemodynamic, histopathological, and immunohistochemical effects of two doses of methylene blue in two rabbit model groups In one group, 5 mg/kg IV was administered, and in another, 20 mg/kg IV was administered following a constant rate infusion (CRI) of 2 mg/kg/h that lasted 6 h. All the groups, including a control group had intestinal ischemia-reperfusion. Immunohistochemical analysis was performed using caspase-3.

**Results:**

During ischemia, hemodynamic depression with reduced perfusion and elevated lactate were observed. During reperfusion, methylene blue (MB) infusion generated an increase in cardiac output due to a positive chronotropic effect, an elevation of preload, and an intense positive inotropic effect. The changes in heart rate and blood pressure were significantly greater in the group in which methylene blue 5 mg/kg IV was administered (MB5) than in the group in which methylene blue 20 mg/kg IV dose was administered (MB20). In addition, lactate and stroke volume variations were significantly reduced, and vascular resistance was significantly elevated in the MB5 group compared with the control group and MB20 group. The MB5 group showed a significant decrease in the intensity of histopathological lesion scores in the intestines and a decrease in caspase-3 areas, in comparison with other groups.

**Conclusions:**

MB infusion produced improvements in hemodynamic parameters in rabbits subjected to intestinal IR, with increased cardiac output and blood pressure. An MB dosage of 5 mg/kg IV administered at a CRI of 2 mg/kg/h exhibited the most protective effect against histopathological damage caused by intestinal ischemia-reperfusion. Further studies with MB in clinical veterinary pathologies are recommended to fully evaluate these findings.

## Background

The intestine is very sensitive to reduction in blood flow. Ischemia can lead to intestinal lesions such as edema, hemorrhages, or mucosal damage. During intestinal ischemia, delivery of oxygen is reduced in the splanchnic compartment and results in depleted adenosine triphosphate levels. This leads to an increase in intracellular calcium levels, initiates anaerobic glycolysis, and activates xanthine oxidase and cell death [1]. In horses, it has been demonstrated that part of this damage after intestinal ischemia is due to alterations in glutathione and S-adenosyl methionine [2]. Recovery of sufficient perfusion may facilitate correction of these changes. Depending on the duration of the ischemic period, reperfusion may result in delivery of toxic oxygen metabolites and free radicals created from hypoxanthine and ß-actin, which may be more severe than those during the ischemic period [1, 3, 4].

The intestinal ischemia-reperfusion (IR) period is therefore associated with multiple adverse effects as follows: increased vascular permeability due to infiltration and adhesion of granulocytes, delivery of proinflammatory cytokines, and production of oxygen radicals and other reactive oxygen species [5]. These microcirculatory effects together lead to hemodynamic alterations and shock which, in many cases, are refractory to commonly used vasoconstrictor drugs [6].

One of the different therapeutic strategies used in human medicine to avoid the negative effects of IR is administration of methylene blue (MB). Although the mechanism of action of MB is complex and is not entirely clear, it can mitigate against some microcirculatory effects. MB has been shown to inhibit the synthesis of the superoxide anion via xanthine oxidase, reduce levels of cGMP by inhibiting guanylate cyclase and nitric oxide synthase, and block the action of nitric oxide [7, 8]. MB has demonstrated a protective effect following ischemia reperfusion in different organs [9–12]. Variable results have been reported on the use of MB in intestinal IR [8, 12–14]. Differences in results in the intestine and in other organs could be due to varying doses or application times [13]. It may also be due to the fact that the intestine is one of the first organs to be affected in a situation of systemic hypo-perfusion. There are no studies that have paid attention to potential uses of MB in veterinary medicine despite of the importance of intestinal IR in common diseases, such as equine colic or gastric dilatation-volvulus (GDV) in dogs.

The primary objectives of this study were to evaluate the hemodynamic and the protective effects of different doses of MB on histopathological lesions in different organs (small intestine, lung, kidneys, and liver) as directed by MB infusion in a rabbit model of intestinal IR syndrome. As a secondary objective, immunohistochemical analysis of caspase-3 was performed in segments of the small intestine to evaluate the presence of apoptosis resulting from ischemia-reperfusion as an early factor to identify this process.

## Results

### Hemodynamic assessment

No differences were observed in any of the parameters at baseline and during the ischemic period among the three groups. The parameters analyzed are shown in Table 1.

**Table 1:** Haemodynamic parameters in control (C) and methylene blue groups (MB5 and MB20)

Variables	Group	Basaline	Ischemia	R60	R120	R240	R360	Reperfusion	
HR (bpm)	C	207 ± 32 ^a^	175 ± 41^a^	173 ± 31	178 ± 27	184 ± 28	190 ± 28	179 ± 29	♯,●
	MB5	200 ± 18 ^a^	182 ± 16 ^a,c^	197 ± 28	209 ± 25	213 ± 24	216 ± 20	207 ± 26 ^c^	♯,†
	MB20	199 ± 23 ^a^	176 ± 23 ^a,c^	183 ± 26	198 ± 29	194 ± 37	200 ± 34	190 ± 30 ^c^	●,†
RR (rpm)	C	35 ± 9	32 ± 8	36 ± 12	328	3510	3510	34 ± 10	♯,●
	MB5	37 ± 10	35 ± 5 ^c^	41 ± 8	436	4810	4610	43 ± 9 ^c^	♯
	MB20	34 ± 7	33 ± 8 ^c^	35 ± 8	4013	4212	4514	40 ± 12 ^c^	●
MAP (mmHg)	C	64 ± 6 ^a,b^	41 ± 13 ^a^	41 ± 11	42 ± 11	41 ± 10	38 ± 11	40 ± 10 ^b^	♯,●
	MB5	66 ± 15 ^a^	46 ± 13 ^a,c^	65 ± 15	58 ± 13	56 ± 13	55 ± 12	59 ± 14 ^c^	♯,†
	MB20	61 ± 14 ª^,b^	47 ± 10 ^a^	50 ± 9	45 ± 9	45 ± 7	46 ± 8	47 ± 9^b^	●,†
SAP (mmHg)	C	84 ± 12 ^a,b^	52 ± 15 ^a^	56 ± 14	59 ± 12	56 ± 12	52 ± 13	56 ± 13 ^b^	♯,●
	MB5	89 ± 23 ^a^	60 ± 18 ^a,c^	95 ± 21	84 ± 15	79 ± 14	75 ± 14	85 ± 18 ^c^	♯,†
	MB20	83 ± 19 ^a^	62 ± 17 ^a^	78 ± 17	70 ± 18	66 ± 14	69 ± 14	72 ± 17	●,†
DAP (mmHg)	C	51 ± 6 ^a,b^	35 ± 11 ^a^	32 ± 9	33 ± 10	33 ± 8	30 ± 9	31 ± 9 ^b^	♯,●
	MB5	52 ± 14 ^a^	39 ± 11 ^a,c^	49 ± 12	45 ± 11	42 ± 10	42 ± 10	45 ± 11 ^c^	♯,†
	MB20	45 ± 13 ^a,b^	37 ± 10 ^a^	35 ± 9	33 ± 7	35 ± 6	37 ± 9	36 ± 6 ^b^	†,●
CI (mL/min/kg)	C	177 ± 52 ^a,b^	115 ± 53 ^a^	124 ± 49	126 ± 28	110 ± 16	127 ± 27	123 ± 39 ^b^	♯,●
	MB5	190 ± 33 ^a^	103 ± 29 ^a,c^	172 ± 47	191 ± 53	186 ± 51	183 ± 45	182 ± 50 ^c^	♯
	MB20	189 ± 39 ^a^	111 ± 32 ^a,c^	185 ± 68	168 ± 56	165 ± 60	177 ± 73	175 ± 63 ^c^	●
dPmx (mmHg/s)	C	888 ± 363 ^a,b^	488 ± 161 ^a^	628 ± 266	605 ± 274	535 ± 158	450 ± 128	584 ± 250 ^b^	♯,●
	MB5	941 ± 193 ^a^	558 ± 237 ^a,c^	1244 ± 357	1072 ± 253	930 ± 256	918 ± 194	1080 ± 314 ^c^	♯
	MB20	931 ± 276 ^a^	577 ± 140 ^a,c^	1218 ± 403	916 ± 463	874 ± 390	1017 ± 405	1031 ± 442 ^c^	●
SVI (mL/beat/kg)	C	0.88 ± 0.31 ^a^	0.68 ± 0.30 ^a^	0.73 ± 0.32	0.71 ± 0.18	0.55 ± 0.17	0.62 ± 0.16	0.69 ± 0.26	♯,●
	MB5	0.95 ± 0.15 ^a^	0.54 ± 0.13 ^a,c^	0.93 ± 0.34	0.98 ± 0.34	0.92 ± 0.30	0.89 ± 0.32	0.94 ± 0.32 ^c^	♯
	MB20	0.98 ± 0.26 ^a^	0.65 ± 0.20 ^a,c^	1.00 ± 0.36	0.83 ± 0.20	0.82 ± 0.22	0.89 ± 0.25	0.91 ± 0.30 ^c^	●
SVRI (mmHg/mL/min/kg)	C	0.37 ± 0.10 ^a^	0.40 ± 0.18 ^a,c^	0.33 ± 0.13	0.30 ± 0.08	0.32 ± 0.07	0.25 ± 0.1	0.30 ± 0.10 ^c^	♯
	MB5	0.33 ± 0.09 ^a^	0.44 ± 0.15 ^a,c^	0.34 ± 0.07	0.32 ± 0.06	0.30 ± 0.08	0.31 ± 0.09	0.33 ± 0.11 ^c^	♯,†
	MB20	0.31 ± 0.11 ^a^	0.42 ± 0.13 ^a,c^	0.26 ± 0.10	0.27 ± 0.06	0.32 ± 0.07	0.30 ± 0.19	0.28 ± 0.09 ^c^	†
CaO_2_ (mL/dL)	C	16.4 ± 1.2 ^a,b^	14.2 ± 1.7 ^a^	15.1 ± 1.6	14.8 ± 1.4	13.8 ± 0.9	12.1 ± 1.9	14.8 ± 2.1 ^b^	
	MB5	15.7 ± 1.8 ^a,b^	14.7 ± 1.5 ^a^	15.6 ± 1.5	14.8 ± 1.5	13.9 ± 1.7	13.5 ± 1.9	14.4 ± 2.1 ^b^	†
	MB20	16.1 ± 1.1 ^a,b^	14.1 ± 2.5 ^a^	13.8 ± 2.6	13.1 ± 2.2	12.7 ± 2.5	12.3 ± 2.7	13.3 ± 2.6 ^b^	†
DO_2_ (mL/min/kg)	C	29.3 ± 8.3 ^a,b^	15.9 ± 8.8 ^a^	19.2 ± 5.9	16.2 ± 4.2	14.7 ± 4.1	14.8 ± 3.0	16.4 ± 4.6 ^b^	♯,●
	MB5	29.5 ± 7.9 ^a^	15.7 ± 7.1 ^a,c^	25.7 ± 6.4	27.0 ± 4.9	24.8 ± 6.7	20.0 ± 7.4	24.4 ± 6.3 ^c^	♯
	MB20	30.1 ± 8.6 ^a^	15.6 ± 4.5 ^a,c^	28.5 ± 13.9	24.5 ± 5.6	21.9 ± 8.2	19.7 ± 8.5	23.6 ± 10.5 ^c^	●
VVS (%)	C	12 ± 2 ^a,b^	22 ± 7 ^a^	19 ± 5	21 ± 6	21 ± 5	24 ± 7	20 ± 5 ^b^	♯
	MB5	10 ± 3 ^a^	19 ± 6 ^a,c^	15 ± 4	15 ± 6	13 ± 5	12 ± 3	14 ± 5 ^c^	♯,†
	MB20	12 ± 3 ^a,b^	19 ± 5 ^a^	17 ± 6	17 ± 8	19 ± 9	19 ± 10	18 ± 8 ^b^	†
Lactate (mmol/L)	C	2.8 ± 1.1 ^a,b^	5.6 ± 1.8 ^a^	7.7 ± 2.0	6.6 ± 2.4	6.0 ± 1.7	5.7 ± 1.9	6.9 ± 2.6 ^b^	♯
	MB5	2.4 ± 1.0 ^a,b^	4.9 ± 1.3 ^a,^	5.5 ± 1.2	4.7 ± 1.7	4.3 ± 1.4	4.5 ± 1.4	4.8 ± 1.2 ^b^	♯,†
	MB20	2.8 ± 0.9 ^a,b^	5.2 ± 1.9^a^	6.3 ± 2.1	6.4 ± 3.1	6.0 ± 3.0	5.5 ± 2.4	6.1 ± 2.4 ^b^	†
Hemoglobine (gr/dL)	C	12.3 ± 1.1 ^a,b^	10.4 ± 1.4 ^a^	10.9 ± 1.6	10.7 ± 1.5	9.2 ± 1.6	8.9 ± 1.4	10.3 ± 1.5 ^b^	
	MB5	11.8 ± 1.3 ^a,b^	10.6 ± 1.1 ^a^	11.7 ± 1.3	11.2 ± 1.5	10.1 ± 1.7	10.0 ± 1.4	10.9 ± 1.4 ^b^	
	MB20	12.2 ± 1.2 ^a,b^	10.5 ± 1.8 ^a^	10.8 ± 2.0	10.1 ± 1.7	9.9 ± 1.8	9.7 ± 1.9	10.3 ± 1.4 ^b^	

Legend: Data are expressed as mean ± SD. The reperfusion column represents the average value of the different periods of reperfusion

^a^ Significant difference (*P* < 0.05) between baseline and ischemia. ^b^ Significant difference (*P* < 0.05) between baseline and reperfusion. ^c^ Significant difference (*P* < 0.05) between reperfusion and ischemia

♯. Significant difference (*P* < 0.05) between Control and MB5 in reperfusion. ● Significant difference (*P* < 0.05) between Control and MB20 in reperfusion.

†. Significant difference (*p* < 0.05) between MB5 and MB20 in reperfusion

During the ischemic period, a significant hemodynamic depression with a drop in cardic index (CI) (− 86 mL/min/kg: CI 95% -105/− 67 mL/min/kg*; p* = 0.001) was observed due to a reduction of preload (stroke volume index (SVI): − 0.35 mL/beat/kg: CI 95% -0.37/− 0.24 mL/beat/kg; *p* = 0.001), contractility (dPmx: − 328 mmHg/s: CI 95% -444/− 212 mmHg/s; *p* = 0.001), and elevation of afterload (systemic vascular resistance index (SVRI): 0.09 mmHg/mL/min/kg: CI 95% 0.02/0.15 mmHg/mL/min/kg; *p* = 0.005). Moreover, this situation was accompanied by a reduction in oxygenation (delivery of oxygen (DO_2_): − 15.3 mL/min/kg: CI 95% -18.4/− 12.1 mL/min/kg; *p* = 0.001), significant elevations of lactate (2.6 mmol/L: CI 95% 2.0/3.1 mmol/L; *p* = 0.001), and stroke volume variation (SVV) (7%: CI 95% 5/10%; *p* = 0.001) in all animals.

**Fig. 1:**
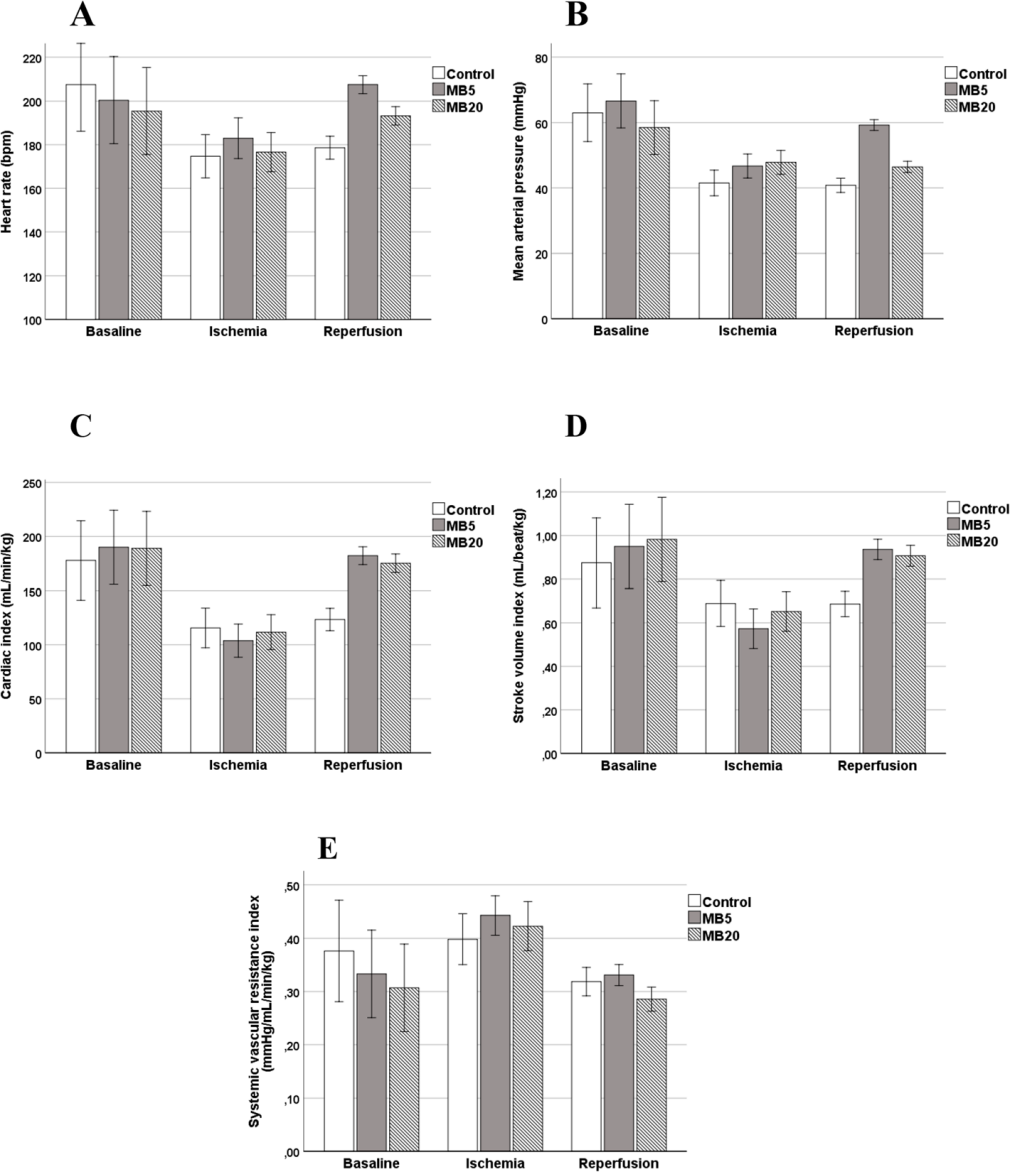
Outcome of main hemodynamic parameters in control, MB5 and MB20 groups in baseline, ischemic and reperfusion periods. Legend: In MB5 group, a significant increase of heart rate, mean arterial pressure, cardiac index and stroke volume index were observed in reperfusion period in comparison to ischemic period. Moreover, a reduction of systemic vascular resistance index was detected. Similar findings were observed in MB20 although less intense than MB5. The changes due to ischemia were not modified in control group during reperfusion period, with the exception of systemic vascular resistance index

During the reperfusion period in the control group, no significant changes were detected in hemodynamic parameters such as heart rate (HR) (*p* = 0.999), mean arterial pressure (MAP) (*p* = 0.998), cardiac index (CI) (*p* = 0.999), dPmx (*p* = 0.372), SVI (*p* = 0.998) or DO_2_ (*p* = 0.999). However, in the MB groups, an improvement in CI was observed in both cases, particularly due to elevation of preload and HR. In MB20, statistically significant increases in HR (17 bpm: CI95% 4–29 bpm; *p* = 0.005), CI (64 mL/min/kg: CI 95% 27/89 mL/min/kg *p* = 0.001), dPmx (385 mmHg/s: CI 95% 207/563 mmHg/s; *p* = 0.001), SVI (0.25 mL/beat/kg: CI 95% 0.13/0.38 mL/beat/kg; *p* = 0.001), DO_2_ (9.7 mL/min/kg: IC95% 3.3/16.0 mL/min/kg; *p* = 0.001), and reduction of SVRI (− 0.14 mmHg/mL/min/kg: CI95% -0.19/− 0.08; *p* = 0.001) were observed in comparison with the ischemic period. Similar findings were observed in the MB5 group, although the hemodynamic improvement was more evident with a significant increase in HR (25 bpm: CI 95% 14–35 bpm; *p* = 0.001), CI (85 mL/min/kg: CI 95% 67/119 mL/min/kg *p* = 0.001), dPmx (520 mmHg/s: CI 95% 395/647 mmHg/s; *p* = 0.001), SVI (0.45 mL/beat/kg: CI 95% 0.36/0.53 mL/beat/kg; *p* = 0.001), DO_2_ (10.2 mL/min/kg: CI 95% 6.3/14.3 mL/min/kg; *p* = 0.001), and a reduction of SVRI (− 0.11 mmHg/mL/min/kg: CI 95% -0.16/− 0.06; *p* = 0.001). Moreover, in MB5, a significant increase in blood pressure (MAP: 13 mmHg: CI 95% 7/18 mmHg; *p* = 0.001) was detected. The hemodynamic changes were significantly greater in the MB5 than in the MB20 group (Fig. 1). In addition, the elevation in SVRI and the reduction of lactate and SVV were significantly greater in the MB5 group than in the control and MB20 groups.

### Histopathological study

A histopathological study was conducted in three different segments of the small intestine: duodenum, jejunum, and ileum. A summary of the results is shown in Table 2.

**Table 2:** Values of histopathological analysis in control and methylene blue groups (MB5 and MB20)

Organ	Control	MB5	MB20
Duodenum	4 (2.8–6.8) ^a^	1 (0.5–1.5) ^a^	3 (2–4)
Jejunum	5.5 (4–6.5) ^a^	2 (0.6–2.4) ^a,b^	4 (4–6) ^b^
Ileum	4 (3–4.5) ^a^	1.25 (0.5–3.4) ^a,b^	4 (3–5) ^b^
Lung^c^	2.5 (1–3)	2.5 (2–3)	2.5 (1–3)
Neutrophils infiltrate^d^	2 (1.5–3)	2(2–2.5)	2 (1.8–2.8)
Liver^e^	2 (1.5–3)	2 (1.6–2.5)	1.5 (1–3)
Kidney^f^	2.5 (2–3)	2.75 (2.5–3)	2.5 (1.9–2.6)

Legend: Data are expressed as median (P_25_–P_75_)

^a^. Significant difference (*P* < 0.05) between MB5 and control group

^b^. Significant difference (p < 0.05) between MB5 and MB20

Scoring system followed for lung, neutrophils infiltrate, liver and kidney (more details in methods section):

^c^0 (absence of lesions). 1 slight (focal lesions), 2 moderate (multifocal lesions), and 3 severe (diffuse lesions)

^d^1 occasional (0–5 cells), 2 slight (6–15 cells), 3 moderate (16–30 cells), and 4 severe (> 30 cells)

^e^0 (normal), 1 (slight), 2 (moderate); 3 (severe) to 4 (very severe)

^f^0 (normal), 1 (slight; 0–50 vacuoles), 2 (moderate; 50–-100) and 3 (severe; > 100)

The most severe histopathological lesions were observed in the control group, with the presence of disepithelized villi and tips as well as dilated capillaries. In some areas, erosion or complete loss of lamina propria was found and crypt layer injury and transmural infarcts were seen in two animals. Mild to severe lesions with extension into the lamina propria with moderate erosion and, in some areas, massive erosion of villi with some disepithelized tips were detected in the MB20 group. The severity of lesions in MB20 meant that in comparison with the control group, no significant differences were observed in any intestinal sections. However, the damage was greater in the group without treatment, with a crypt layer injury as well as more frequent transmucosal infarction, particularly in the jejunum.

In contrast, lesions in the MB5 group consisted of subepithelial dilation at the villous tips, with minimal alterations and no crypt layer injury of transmucosal infarction. Four animals in the MB5 group showed a score of ≤1, which determined that significant differences were detected between MB5 and the control group in the duodenum (2.75: CI 95% 1.5/6; *p* = 0.021), jejunum (3.5: CI 95% 3/5; *p* = 0.002), and ileum (2.5: CI 95% 0.5/3.5; *p* = 0.042). Moreover, a protective effect of MB5 was also detected in the jejunum (3: CI 95% 2/4; *p* = 0.018) and ileum (2.5: CI 95% 0.5/4; *p* = 0.037), unlike in the MB20 group.

In the three groups that were tested, similar lesions were seen in the lungs. The statistical analysis showed no differences between groups (*p* = 0.833). In all groups moderate to severe changes were observed, with multifocal areas of interstitial edema with peribronchial pattern. Multifocal distribution of inflammatory infiltrate composed of neutrophils within capillaries was also present. In relation to neutrophil infiltrates, all groups showed moderate infiltration of this cellular subset. The MB5 group showed a slight decrease in neutrophil infiltrates in comparison to the control and MB20 groups; however, this decrease was not significant (*p* = 0.513). No statistical differences were found between groups with respect to the hepatic lesions (*p* = 0.586). These parameters evaluated were centrolobullar degeneration, hepatocytes damages (nuclear damage, presence of binuclear hepatocytes) as well as the presence of inflammatory infiltrate of neutrophils within sinusoids. In the kidneys, we observed the presence of vacuoles within the epithelial cells of renal tubules. The results showed a moderate to intense score in all groups tested, without statistical differences between the groups (*p* = 0.400).

### Immunohistochemical study

**Fig. 2:**
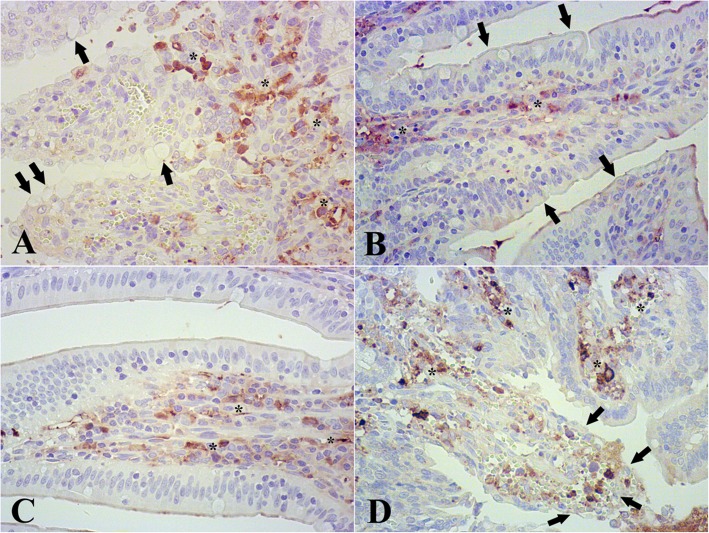
Immunohistochemistry pictures of control group (**a**), negative control (**b**), MB5 group (**c**) and MB20 group (**d**). Legend: In control group (positive control) numerous caspase 3+ areas (asterisk), as well as vacuolitation of epithelial cells (arrows). In negative control scarce caspase 3+ areas (asterisk) were found; villi and epithelial cells without histopathological changes (arrows). MB5 group showed few caspase 3+ areas (asterisk). MB20 group showed severe lesions composed of erosion of villi, disepithelized tips and high subepithelial space (arrows). In this group there was a severe expression of caspase 3+ areas (asterisk). Magnification 400x

**Fig. 3:**
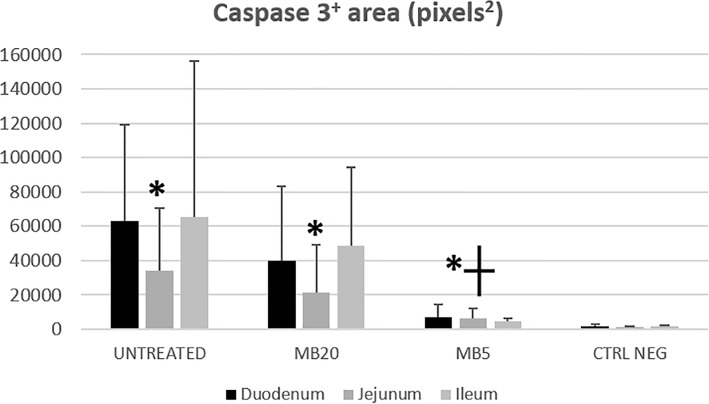
Caspase 3^+^ areas (pixels^2^) by immunohistochemistry in control, MB5 and MB20 groups Legend: Expressed as mean ± SD. Asterisk: significant increase of expression in all groups affected of ischemia/reperfusion in comparison with negative control group. Cross: significant decrease in caspase 3+ areas found in MB5 group respect to MB20 group and untreated groups

The immunohistochemical study revealed a significant increase in caspase-3 areas in MB5 and MB20 groups, and in the positive control group in comparison with the negative control group (Fig. 2). When the caspase-3 areas were compared to the groups where ischemia reperfusion was applied, there were no statistical differences between the positive control group and the MB20 group (*p* = 0.795). However, the MB5 group showed a significant decrease in caspase-3 areas in comparison to the MB20 group (42,005: CI 95% 14,250/69671; *p* = 0.003) and the positive control group (49,384: CI 95% 6160/92606; *p* = 0.023) (Fig. 3).

## Discussion

In human medicine the use of MB for different types of shock has been demonstrated [15], but there is a lack of research on its use in veterinary medicine. Improvements in various hemodynamic parameters associated with the administration of MB have been observed previously in several studies. The mechanism of vascular action of this drug is however, controversial. In septic shock, a bolus of MB at 1 mg/kg produces a transient elevation of MAP and systemic vascular resistance, although without changes in cardiac output (CO), HR, or pulmonary artery occlusion pressure [16]. A similar finding has been observed in patients with perforation peritonitis after administration of 2 mg/kg MB [17]. An elevation of MAP and CO without changes in SVR and CVP has been observed in in humans undergoing orthotopic liver transplantation after 1.5 mg/kg of MB administered prior to reperfusion [18]. These findings are partially in agreement with the present study since in both the groups, MB5 and MB20, an increase in CO due to a chronotropic and inotropic effect, with elevation of preload and without changes in afterload was detected. This cardiovascular improvement resulted in an elevation of DO_2_, and in MB5, along with a reduction in lactate, which would imply an improvement in the degree of cellular perfusion of tissues.

An in vitro study using endothelial cells demonstrated that the immunomodulatory effect produced by MB was influenced by dose and time [19]. This finding might also be applicable to in vivo conditions and may explain the differences between the two doses of MB in our study. The most common dose used in humans is 2 mg/kg, which is used to treat methemoglobinemia in humans; however, because this dose has not been proven to be more effective than other doses [20]. The hemodynamic changes were significantly greater in the MB5 group, especially on blood pressure, HR, and afterload, with a reduction in VVS. It is possible that the minor hemodynamic effect observed in the MB20 group is the result of a high dosage. That could alter the perfusion of the tissues and result in more effective reductions in lactate and SVV than in the MB5 group. These findings are in agreement with a study in which different doses of MB were infused in humans with septic shock, and the authors observed that high doses could damage the splanchnic perfusion, as reflected by the elevation of gastric tonometry [21]. Plasma values of MB start to reduce after forty minutes of one single bolus. For this reason, an infusion is preferable to maintain the hemodynamic effect in a sustained manner [20, 22]. Although 5 mg/kg MB administered at a CRI of 2 mg/kg/h was shown to be most effective in our study, further studies are necessary to determine the optimal dosage of MB in clinical conditions in dogs or horses.

Different studies have shown that the administration of MB prior to reperfusion is the most effective moment to treat IR [10, 18, 20]. In this study, we administered MB 10 min before the start of the reperfusion period. Although we observed similar findings in the first minutes after reperfusion in all groups, the recovery of hemodynamic parameters was faster in the MB groups. We interpret that early administration of MB in IR cases is more beneficial than delaying administration until the onset of a critical hypodynamic status after reperfusion. We believe that the use of MB prior to derotation of the stomach in GDV or the intestine in equine colic would be beneficial to buffer the effect of IR in these pathologies, although further clinical studies are necessary to evaluate this advantage.

Despite the protective effect of MB on the brain, lung, or kidneys after IR [9–11, 23], the effects on the intestine have not been clearly documented in previous studies. In a rodent model of intestinal IR, intraperitoneal administration of MB did not reduce the inflammatory lesions in the small bowel [12]. A similar outcome was observed when MB was combined with pentoxyphylline and lidocaine [24].

In organs with slow recovery after hypoperfusion, such as the intestine, these differences could be due to the dose of MB or evaluation time after reperfusion [13]. For this reason, in the present study, two different doses were evaluated, and the reperfusion period was 6 h. This period was longer than that used in previous studies with results without a protective effect [12, 13]. With this design, MB5 showed significantly decreased lesions in the small intestinal segments (duodenum, jejunum, and ileum) compared to the MB20 and control groups, which suggests a protective action of MB5 in intestinal IR.

It has been reported that intestinal IR injury is related to an increase of apoptosis in this location. This might suggest a crucial role in the pathogenesis of the process [25–27]. Authors of a recent paper have concluded that MB reduces the apoptosis and inflammatory response in renal IR [28]. Moreover, MB restores the mitochondrial function of the liver after intestinal IR [14]. In the present study, caspase-3 showed a significant increase in injured groups (treated and untreated). Values were significantly lower in MB5 in comparison to the MB20 and control groups. Since the IR injury protocol was the same for all groups, the decrease of caspase- 3 areas in the MB5 group suggests a protective role in preventing the histopathological damage and apoptosis caused by intestinal IR.

The importance of gastrointestinal IR in veterinary medicine is expansive because common diseases such as GDV or equine colic are frequent and can have extensive consequences [29, 30]. Although surgery is an essential element in the treatment of these pathologies, the use of different drugs to improve the recovery of the patients has been and should continue to be evaluated. Lidocaine has demonstrated a protective effect on the smooth muscle in horses with intestinal IR injury [31]. Lidocaine use has not shown influence on survival rates [32]. The selective cyclooxygenase-2 inhibitor robenacoxib helps in recovering the functionality of the jejunal mucosa in horses experiencing ischemia [33]. Despite the interest in using MB to treat IR in human medicine, no studies have been conducted in veterinary medicine to elucidate the hemodynamic action or protective effect of this drug after intestinal IR. The present research carried out in a rabbit model shows the beneficial effect of MB infusion to treat intestinal IR and supports the need for additional studies to determine the utility of this drug in veterinary clinical conditions.

## Conclusions

Infusion of MB produced improvements in hemodynamic parameters in rabbits subjected to intestinal IR by increasing cardiac output due to chronotropic and inotropic effects. Elevation in preload and consequent elevations in blood pressure and delivery of oxygen were also seen. A dosage of 5 mg/kg IV MB administered at a CRI of 2 mg/kg/h exhibited the most protective effect against histopathological damage caused by intestinal IR. Immunohistochemical study of caspase-3 of the small intestine will be useful to evaluate the presence of apoptosis after intestinal ischemia-reperfusion. Additional studies to determine the utility of this drug in veterinary clinical conditions are necessary.

## Methods

### Animals

Twenty-one New Zealand White rabbits MDL (10 male and 11 female: 1.7 ± 0.3 years), weighing 4.3 ± 1.2 kg, were used in this study. The animals were acquired from a breeding center animal for research animals (Granja San Bernando SL, Spain), they were healthy, and had not experienced previous infectious or general diseases. The experiment was approved by the Bioethical Committee of the University of Córdoba (NRG/6897) and conducted in accordance with the European (2010/63/UE) and national (RD 1201/2005) directives on animal experimentation. Study was carried out following the ARRIVE guidelines for the reporting of animal experiments. A prospective power analysis was used to determine the number of animals required to document differences in CI between the three groups in reperfusion period. The results of this analysis confirmed that no more than 21 rabbits were needed (7 in each group) considering an α error of 0.05, a β error of 0.80, a SD of 35 and an effect size of 0.75.

### Anesthetic protocol and monitoring

In each animal, the right ear was clipped and a lidocaine-prilocaine ointment (EMLA cream, AstraZeneca, Spain) was applied on the convex part of the pinna over the auricular vessels. Fifteen minutes later, the ear was aseptically prepared with chlorhexidine (Desinclor 5%, AGB, Spain) and a catheter (Vasocan 22 G, B. Braun, Spain) inserted in the lateral ear vein.

Morphine (0.3 mg/kg IM; Morphine 2%, B. Braun, Spain) and medetomidine (15 μg/kg IV; Domitor, Vetoquinol, UK) were administered, and after 10 min, induction was performed with intravenous (IV) administered propofol to effect (Propofol-Lipuro, B. Braun, Spain). When corneal and palpebral reflexes were absent, tracheal intubation was performed using a cuffed endotracheal tube. Anesthesia was maintained using isoflurane (Isoflo, Abbott, UK) delivered in a mixture of 50% oxygen and air with a semi-closed rebreathing system.

A catheter was inserted in both the right external jugular vein (Introcan 20 G, B. Braun, Spain) and the right femoral artery (Pulsiocath 3 F, Pulsion Medical Systems, Germany), which was facilitated by small surgical incisions. At this point, blood samples were obtained for analysis of baseline values. Throughout the study period, a crystalloid solution (Lactated Ringers solution; B. Braun Vet Care, Spain) was IV administered at 5 mL/kg/h via the lateral ear vein, mechanical ventilation was applied to maintain normocapnia, body temperature was maintained at 37 °C by using a forced-air warming blanket (Equator, Smiths Medical ASP, UK), and morphine administration was repeated every 4 h for analgesia.

### Design of study and surgical procedure

The animal was positioned in dorsal recumbency and the abdominal area was clipped and aseptically prepared with chlorhexidine. A midline celiotomy was performed, and the cranial mesenteric artery and portal vein were located and occluded with bulldog clamps. Vessel occlusion was confirmed by observing intestinal congestion, intestinal arterial pulselessness, and increased peristalsis, and this was the start of the 60 min ischemic period. After occlusion, the abdominal cavity was closed temporarily and covered with a surgical drape (Opsite, Smith & Nephew, UK) to avoid contamination and heat loss.

To carry out the study, the rabbits were divided randomly (www.randomizer.org) into three groups, each composed of seven rabbits: a positive control group and two treated groups (groups MB5 and MB20). The treatments began 10 min before the end of the ischemic period, during which time the abdominal cavity was re-opened, and the clamps were removed. The 60 min ischemic period was followed by a reperfusion period of 360 min. In the MB5 group, each animal received an initial MB (Methylene Blue 1%, Far. Luis Corbí, Spain) bolus of 5 mg/kg IV over 10 min, followed by a constant rate infusion (CRI) of MB at 2 mg/kg/h during the reperfusion period. In the MB20 group, each animal received an initial MB bolus of 20 mg/kg over 10 min, followed by a CRI of MB at 2 mg/kg/h during the reperfusion period. Each animal in the control group received an initial saline bolus of 2 mL/kg followed by CRI of saline with a total volume similar to that of the MB administered in the other groups.

Heart rate (HR), mean arterial pressure (MAP), systolic arterial pressure (SAP), diastolic arterial pressure (DAP), cardiac output (CO), systemic vascular resistance (SVR), stroke volume (SV), stroke volume variation (SVV), and contractility (dPmx) were measured with a PiCCO monitor (PiCCO plus, Pulsion Medical Systems, Germany). Two 5 mL boluses of cold (≤ 8 °C) saline were first administered to the rabbits via the jugular vein for calibration of the instrument. If a high variability was detected between the two assessments, a third bolus was administered. Respiratory rate (RR) was measured using a multiparametric monitor (Anesthesia Monitor, Datex-Ohmeda, GE Healthcare, Chicago, IL, USA). These variables were recorded every 15 min.

Hemoglobin (Hb), lactate, and arterial blood gas measurements (Ciba-Corning 850, Siemens, Germany) were performed using arterial blood samples obtained at baseline, the start of (I0) ischemia, and at 30 (I30) and 50 (I50) min during ischemia, as well as at 60 (R60), 120 (R120), 240 (R240), and 360 (R360) min during reperfusion. Delivery of oxygen (DO_2_) and arterial oxygen content (CaO_2_) were calculated. CO, SV, and SVR were expressed as indexed parameters (i.e. cardiac index, SVI, and SVRI). For analysis, values pertaining to the ischemic period were considered the mean of I0, I30, and I50.

### Tissue samples

Following euthanasia (140 mg/kg IV; Sodium Pentobarbital, Euthasol 400 mg/ml, Ecuphar, Spain) necropsy of the animals was performed. For each animal, tissue samples were taken from the small bowel (duodenum, jejunum, and ileum), lung, kidneys, and liver. The samples were fixed in 10% neutral buffered formalin and embedded in paraffin wax. Then, formalin-fixed and paraffin wax-embedded samples were sectioned into 4 μm sections to perform both the histopathological and immunohistochemical studies. In addition to samples obtaining of the three groups, intestine samples of three healthy rabbits no undergoing to intestinal ischemia reperfusion were obtained to inmunohistoquimical analysis, as negative control group. These three animals were sacrificed for causes unrelated to this study.

### Histopathological study

4 μm-thick sections were stained with hematoxylin and eosin to perform the histopathological study. To evaluate the intestinal lesions, the Park-Chiu score was used [34]. In the lung, we evaluated the degree of interstitial edema and neutrophilic infiltration following the criteria described previously [12]. The scoring was as follows: 0 (absence of lesions). 1 slight (only focal lesions), 2 moderate (multifocal lesions), and 3 severe (diffuse lesions). Infiltration of neutrophils was classified within four categories depending on the mean number of cells observed in 10 fields of view, at a magnification of 200x: 1 occasional (0–5 cells), 2 slight (6–15 cells), 3 moderate (16–30 cells), and 4 severe (> 30 cells). In the kidney, the presence of vacuoles within the epithelial of both proximal and distal convoluted tubules was evaluated. The score was established as 0 (normal), 1 (slight; 0–50 vacuoles), 2 (moderate; 50–-100) and 3 (severe; > 100). For the liver, a classification with four categories from 0 (normal), 1 (slight), 2 (moderate); 3 (severe) to 4 (very severe) was applied. The parameters evaluated were centrolobullar degeneration (where hepatocytes showed moderate lipidosis with the presence of intracytoplasmic well-defined microvesicles with no displacement of the nucleus), hepatocytes injury (slight nuclear peripheral chromatin margination affecting some hepatocytes, binucleated hepatocytes), and the presence of inflammatory infiltrate composed by neutrophils in sinusoids. For all the samples, 10 fields of view were selected randomly at 200x magnification, analyzed independently and evaluated by two pathologists blinded to the group designation.

### Immunohistochemistry and Morphometrical analysis

To conduct the immunohistochemical study, the SignalStain® Cleaved Caspase-3 (Asp175) IHC Detection Kit (Cell Signaling Technology, Danvers, MA, USA) was used. The protocol was performed following the manufacturer’s instructions. Briefly, after three deparaffinization steps followed by four rehydration steps, the antigen unmasking was carried out by immersion in 0.01 M Sodium citrate buffer (pH 6.0). It was necessary to boil the solution and then maintain it at a sub-boiling temperature for 10 min. After cooling the slides in buffer for 30 min, the Peroxidase Quench reagent was applied at room temperature for 25 min. Next, two washes in distilled water were performed and the Blocking Solution reagent was applied to the tissue for 60 min at RT. Then, the primary antibody (Cleaved Caspase-3 #9661) was applied overnight at 4 °C. Afterwards, the Biotinylated Secondary Antibody was applied to the tissue for 25 min at RT. During this time, the Avidin-Biotin-Peroxidase (ABC) complex was prepared. After three washes in Phosphate Buffer Saline/Tween (PBS/T), the ABC complex was applied for 30 min at RT, followed by another three washes of PBS/T. Subsequently, the Substrate-Chromagen (Novared Substrate) was prepared and applied for 2–10 min. Finally, tissue sections were lightly counterstained with Mayer’s hematoxylin, dehydrated, and mounted. Tissue sections in which the specific primary antibody was replaced by the prediluted negative control reagent were used as negative controls.

Using Image J software (National Institute of Health, Bethesda, Maryland, EEUU), caspase-3 immunoreactive areas were calculated from randomly selected fields viewed at 400x magnification. Macros were calibrated for staining intensity to include all immunostained cells. A total of five fields for each small bowel segment were analyzed per slide, per animal, and the caspase 3 areas were calculated. Photomicrographs were taken using an Olympus BX51 photomicroscope at 400x magnification.

### Statistical analysis

The software IBM SPSS Statistics 25.0 was used for statistical analysis. The normality of quantitative parameters was evaluated using the Shapiro-Wilks test. A generalized linear mixed model was applied to detect differences in the hemodynamic parameters according to the treatment (control, MB5, MB20) and phase (baseline, ischemia, reperfusion). If statistical differences were detected, a one-way ANOVA was performed with a Bonferroni or Games-Howell as post hoc tests. Comparisons of the histopathological and immunohistochemical parameters between groups were performed using Kruskal-Wallis test, with Dunn test-Bonferroni’s correction as the post hoc test. Statistical significance was indicated with a *p*-value < 0.05. Quantitative data was shown as the mean ± SD, and ordinal values as median (P_25_–P_75_).

## Data Availability

The datasets used and/or analyzed in the current study are available from the corresponding author on reasonable request.

## Abbreviations

ABC: Avidin-Biotin-Peroxidase
CaO_2_: Arterial oxygen content
cGMP: Cyclic guanosine monophosphate
CI: Cardiac index
CO: Cardiac ouput
CRI: Constant rate infusion
CVP: Central venous pressure
DAP: Diastolic arterial pressure
DO_2_: Delivery of oxygen
dPmx: Contractility
GDV: Gastric dilatation-volvulus
HR: Heart rate
IR: Ischemia reperfusion
MAP: Mean arterial pressure
MB: Methylene blue
PBS/T: Phosphate Buffer Saline/Tween
SAP: Systolic arterial pressure
SVI: Stroke volume index
SVRI: Systemic vascular resistance index
SVV: Stroke volume variation
